# Image dataset of benthic foraminifera in multicorer and gravity corer sediments from north-western Scotland shelf (North Atlantic Ocean)

**DOI:** 10.3897/BDJ.10.e87457

**Published:** 2022-07-22

**Authors:** Liubov Kireenko, Anna Tikhonova, Nina Kozina, Alexander Matul

**Affiliations:** 1 Shirshov Institute of Oceanology, Russian Academy of Sciences, Moscow, Russia Shirshov Institute of Oceanology, Russian Academy of Sciences Moscow Russia

**Keywords:** benthic foraminifera, micropaleontology, Scotland shelf, North Atlantic Ocean, Quaternary sediments

## Abstract

Geological studies in the seas and oceans often give preference to the study of benthic foraminifera, which are a widespread and taxonomically diverse group of shell protozoa. In this paper, we present an extensive image dataset produced during the detailed micropaleontological analysis of 146 samples of bottom sediments collected by multicorer and gravity corer AMK-5656 from the Westray Basin on the north-western Scotland shelf (North Atlantic Ocean). In total, 106 taxa (at species and genera level) of benthic foraminifera were identified and photographed using the high-resolution microscope camera. This dataset can aid as a guide for identification of the benthic foraminiferal taxa at the paleoecological studies, stratigraphic works and interregional paleoceanographic correlation in the North Atlantic Ocean.

## Introduction

The North Atlantic (NA) is one of the key areas of the thermohaline circulation system of currents that transfer the heat, salt, dissolved elements and gases and sedimentary matter to the Subarctic and Arctic regions. This circulation being a part of the large-scale circulation of the World Ocean affects the warming and cooling of the global climate and regional oceanography in the NA and Arctic (Morley et al. 2011). The climatic regime sets the marine environments, in particular, the habitat of the microorganisms which are preserved in the bottom sediments. An environmental imprint in the microfossil assemblages provides information on the modern and past oceanographic, climatic and ecological changes. Benthic foraminifers as calcareous microorganisms are one of the leading microfossil groups in the marine geological studies giving the appropriate biostratigraphic and paleoecological data.

In this article, we present a new extensive dataset of the microphotographic illustrations with taxonomic coverage for 106 benthic foraminiferal taxa (mostly as species and genera) from the latest Pleistocene to the the Holocene sediments in the Westray Basin on the north-western Scotland shelf (NWSS). The sediment material was collected in summer 2018 during the 71^st^ cruise of the research vessel Akademic Mstislav Keldysh (Novigatsky et al. 2019). Laboratory treatment and microscopic analysis followed the standard micropaleontological technique for benthic foraminifera. We used the sediment fraction of > 63 μm to count small foraminiferal tests which can be numerous in the high-latitude sediments.

Previous studies described diverse and abundant benthic foraminiferal microfauna in the modern and the the Holocene sediments on and around the Scotland shelf and provided some microphotographs of the typical species (Lo Giudice Cappelli et al. 2019, Mackensen 1987, Mojtahid et al. 2021). Our aim is to continue a research study on the identification and illustration of the benthic foraminifera in the North Atlantic bottom sediments (Kireenko et al. 2022). We present 17 tables with high-quality microphotographs of 106 identified foraminiferal taxa (species and genera) produced using the Nikon microscope SMZ25, equipped with Nikon camera DS-Fi3 and NIS-Elements D software. This work intends to update existing guides on the benthic foraminifers from the European continental margin (like Murray 2003) potentially helping in the future routine micropaleontological studies in the area.

## Data Description

**Figure 1.**
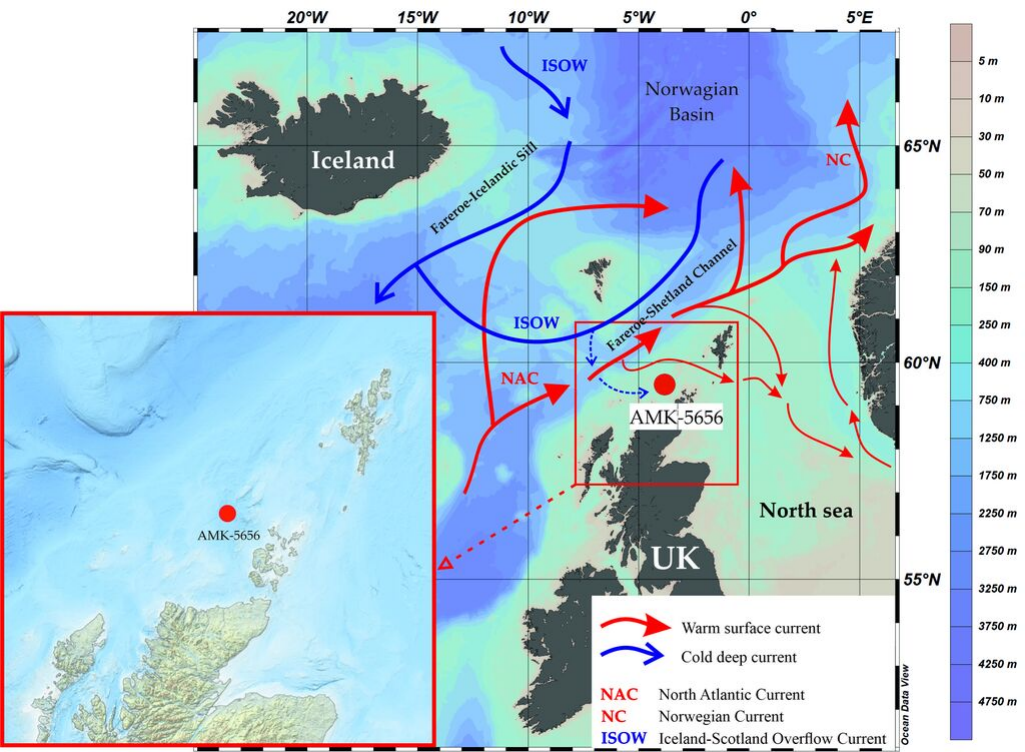
Location of studied sediment cores AMK-5656 MC and GC. Map sources are GEBCO at https://download.gebco.net and EMODnet at https://portal.emodnet-bathymetry.eu.

We studied the benthic foraminiferal microfauna from the multicorer (MC) and gravity corer (GC) sediments on the AMK-5656 station obtained during the 71^st^ cruise of the Russian research vessel Akademik Mstislav Keldysh in summer 2018 (Novigatsky et al. 2019). The location of the AMK-5656 station is the Westray Basin on the NWSS to the northwest of the Orkney Islands (59°29.469’ N, 3°49.783’ W; 157 m depth). The sediments of the MC (18 cm long) and GC (625 cm long) are the alternating foraminiferal light-brown sandy and muddy silts (Fig. 1).

The NWSS is a shallow water region of western North Atlantic Ocean dominated by the warm saline surface water of the North Atlantic Current branch crossing the NWSS from west to east between Orkney and Shetland (Anonymous 2022a). The cold deep water of Iceland-Scotland Overflow Current coming from the north via the Faroe-Iceland Channel can also influence the local bottom environments (Rasmussen et al. 2002, Sejrup et al. 2004). A prevailing sediment type in the Westray Basin is muddy sand or slightly gravelly sandy mud (Anonymous 2022b) which, in general, is similar to what we found in the AMK-5656 MC and GC.

Bio-monitoring studies, based on the living fauna, indicate that the taxonomically diverse benthic foraminifers densely populate the high-latitude shelf areas (Schönfeld et al. 2012). They sensitively react to environmental change and reflect the conditions occurring both at the bottom (factors of direct habitat on and in sediments) and the surface (e.g. phytoplankton production as food source) (Bauch et al. 2001).

Table 1 presents a list of the identified benthic foraminiferal species. We found in the samples of the AMK-5656 MC and GC, 106 taxa all in all identified mostly at species level, partly as genera. Amongst them, four species have the agglutinated shells and the rest are calcareous-secretive. The microphotographs on Figs 2, 3, 4, 5, 6, 7, 8, 9, 10, 11, 12, 13, 14, 15, 16, 17, 18, 19 present high-resolution images of all identified benthic foraminifers showing the shell morphology of every taxa in details from two-three views (apertural, lateral, side, umbilical and spiral).

Studies of the radiocarbon dated sediment cores in Shetland-Orkney area of the NWSS (Bradwell et al. 2021) concluded that the ice-sheet withdrawal from the Westray Basin could occur just before 17.5 calendar ka. After this time level, we can expect starting marine sedimentation in the area. Therefore, our cores AMK-5656 MC and GC may contain records of the deglaciation and the Holocene. The radiocarbon datings on the AMK-5656 cores are not yet ready. There is a prominent change in the main parameters of the sedimentology, geochemistry and benthic foraminifera at the GC core level of 295 cm. Under the level: the content of terrigenous matter increases, CaCO_3_ content is between 25 and 40%, total abundance of benthic foraminifers is low and the infaunal shelf/slope species *Buliminamarginata* and *Fursenkoinafusiformis* have increased concentrations indicating the high fluxes of the total organic carbon and oxygen-depleted conditions (Alve 1994, Eichler et al. 2014). Above the level: opposite distribution of these parameters with a significant rise of CaCO_3_ and total foraminiferal content and sharp decline of the above-mentioned species abundance. Possible explanation of such change could be a transition from deglacial to the Holocene environments, but this will be proven by the radiocarbon dating.

## Methods

Onboard, the retrieved MC and GC sediments were subsampled through every 1 cm and stored in the refrigerator. In the shore laboratory, we analysed 19 samples from the MC (every 1 cm) and 127 samples from the GC (every 5 cm). All samples were freeze-dried, weighed, washed in the distilled water through a sieve with mesh size of 63 µm as recommended in Fatela and Taborda 2002, Klootwijk and Alve 2022, dried and weighed again. We routinely counted 150-300 benthic foraminiferal tests per one sample under the microscope Nikon SMZ800N with a magnification of 80x. The microphotographs were made using the Nikon microscope SMZ25, equipped with Nikon camera DS-Fi3 and NIS-Elements D software. Then, microphotograph tables were edited by the computer software Adobe Photoshop CC 2019. To identify benthic foraminiferal taxa, we used publications by Feyling-Hanssen et al. 1971, Holbourn et al. 2013, Jones 1994, Tikhonova et al. 2019.

## Usage notes

The microphotograph tables with images of benthic foraminifers can be used in the practical micropaleontological work with the modern and Quaternary sediment samples from the high-latitude areas of the North Atlantic. They will help the species identification, description of the foraminiferal assemblages and interpretation of the micropaleontological data for the biostratigraphy and paleoecology.

## Figures and Tables

**Figure 2.**
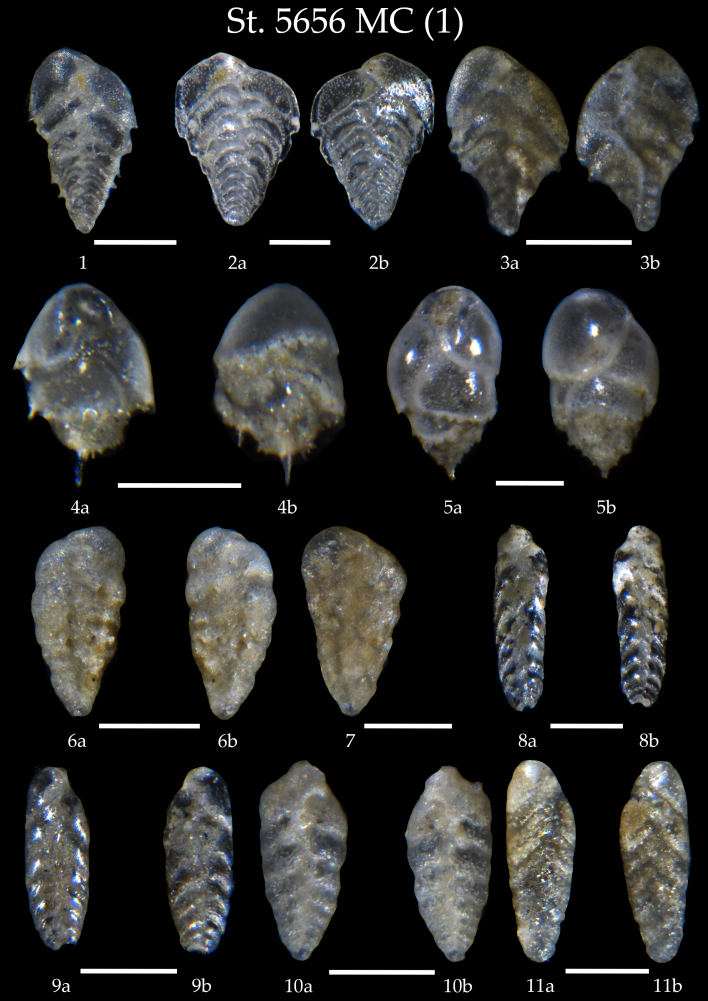
Station 5656 **MC**. **1** *Brizalinapygmaea*; **2** *Brizalinaalata*, **a**,**b** lateral view; **3** *Bolivinatortuosa*; **4** *Buliminaaculeata*, **a** apertural view, **b** lateral view; **5** *Buliminamarginata*, **a** apertural view, **b** lateral view; **6** *Bolivinapseudoplicata*, **6a** side view, **6b** lateral view; **7** *Bolivinapseudoplicata*; **8**,**9** *Bolivinaearlandi*, **a** side view, **b** lateral view; **10**,**11** *Bolivinastriatula*, **a** side view, **b** lateral view. Scale 100 µm.

**Figure 3.**
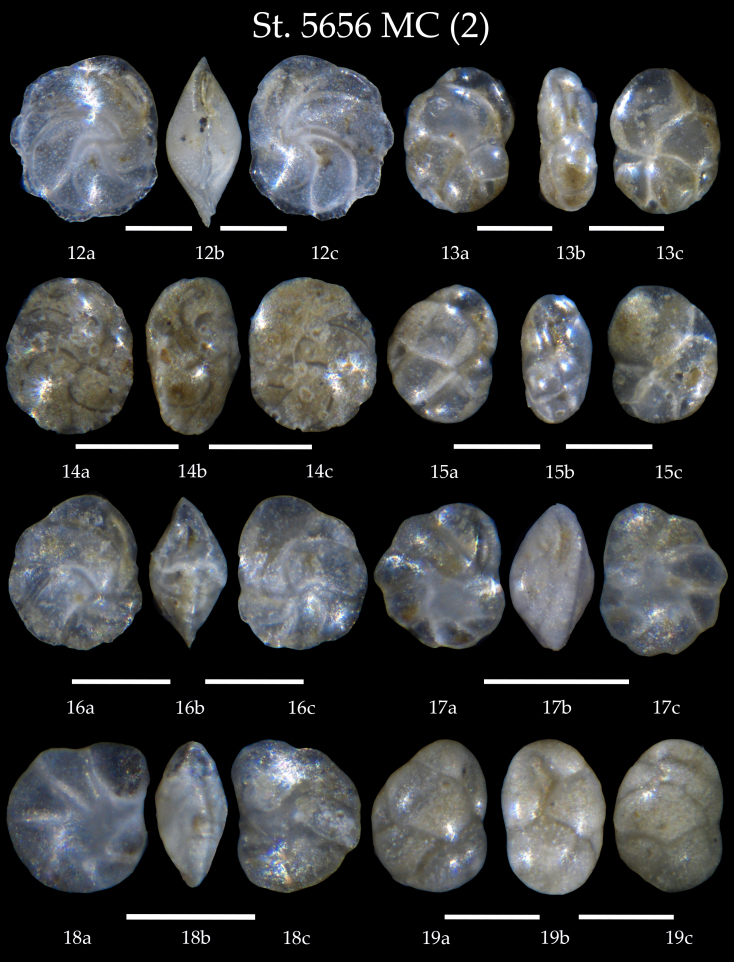
Station 5656 **MC** (continued). **12** *Cassidulinacarinata*, **a, b** apertural view, **c** lateral view; **13** *Cassidulinaobtusa*, **a, b** apertural view, **c** lateral view; **14** *Cassidulinalaevigata*, **a, b** apertural view, **c** lateral view; **15** *Cassidulinareniforme*, **a, b** apertural view, **c** lateral view; **16** *Cassidulina* sp., cf. *C.laevigata*, **a, b** apertural view, **c** lateral view; **17** *Cassidulinateretis*, **a, b** apertural view, **c** lateral view; **18** *Islandiellanorcrossi*, **a, b** apertural view, **c** lateral view; **19** *Globocassidulinasubglobosa*, **a** side view, **b** apertural view, **c** lateral view. Scale 100 µm.

**Figure 4.**
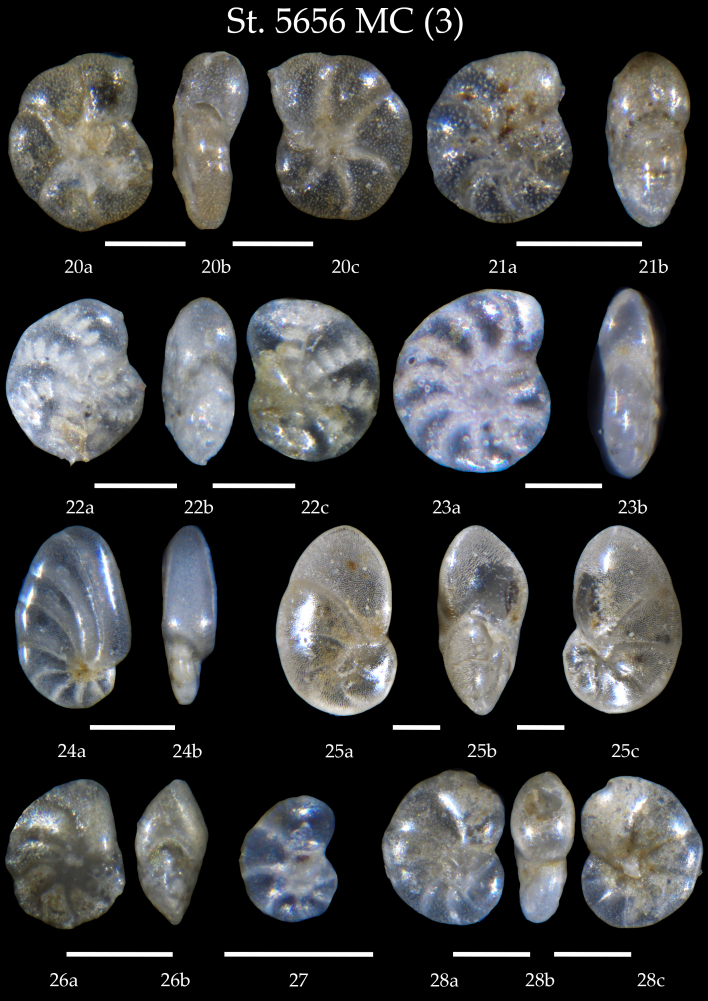
Station 5656 **MC** (continued). **20** *Astrononiongallowayi*, **a** side view, **b** apertural view, **c** lateral view; **21** *Elphidiumclavatum*, **a** side view, **b** apertural view; **22** *Elphidiumearlandi*, **a** side view, **b** apertural view, **c** lateral view; **23** *Elphidiumgerthi*, **a** side view, **b** apertural view; **24** *Nonionoidesturgidus*, **a** side view, **b** apertural view; **25** *Nonionellairidea*, **a** spiral view, **b** apertural view, **c** umbilical view; **26** *Nonionpauperatum*, **a** side view, **b** apertural view; **27** *Nonionellaauricula*; **28** *Nonion* sp. cf. *N.faba*, **a** spiral view, **b** apertural view, **c** umbilical view. Scale 100 µm.

**Figure 5.**
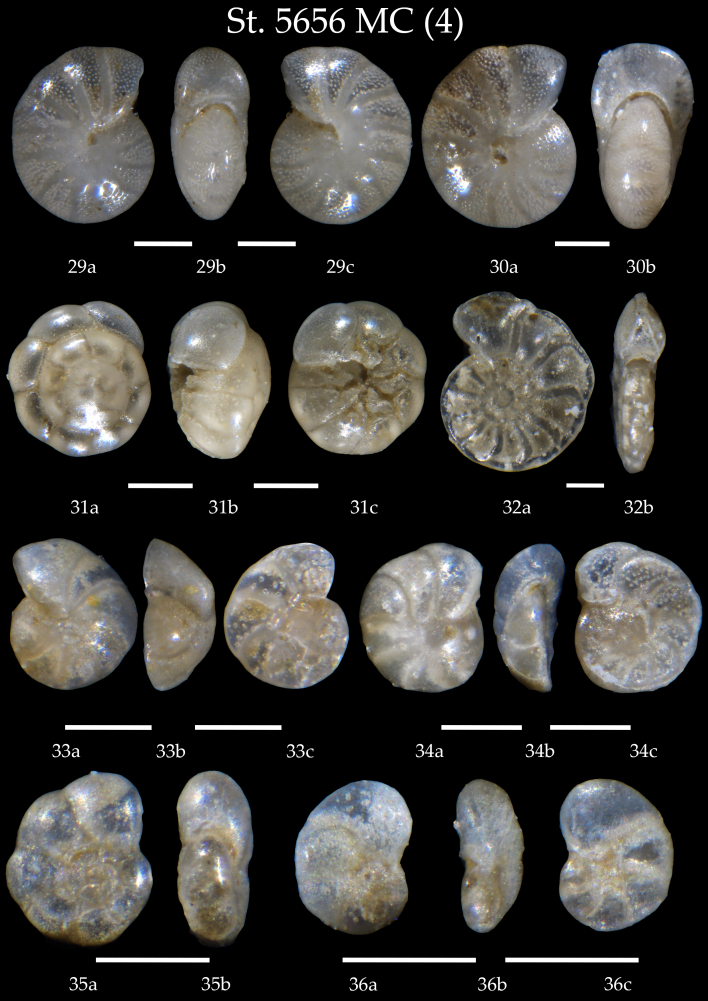
Station 5656 **MС** (continued). **29** *Melonisbarleeanus*, **a** side view, **b** apertural view, **c** lateral view; **30** *Melonispompilioides*, **a** side view, **b** apertural view; **31** *Ammoniafalsobeccarii*, **a** spiral view, **b** apertural view, **c** umbilical view; **32** *Hyalineabalthica*, **a** side view, **b** apertural view; **33** *Cibicidesrefulgens*, **a** spiral view, **b** apertural view, **c** umbilical view; **34** *Cibicideslobatulus*, **a** spiral view, **b** apertural view, **c** umbilical view; **35** *Epistomaroidespolystomelloides*, **a** side view, **b** apertural view; **36** *Planulinaariminensis*, **a** spiral view, **b** apertural view, **c** umbilical view. Scale 100 µm.

**Figure 6.**
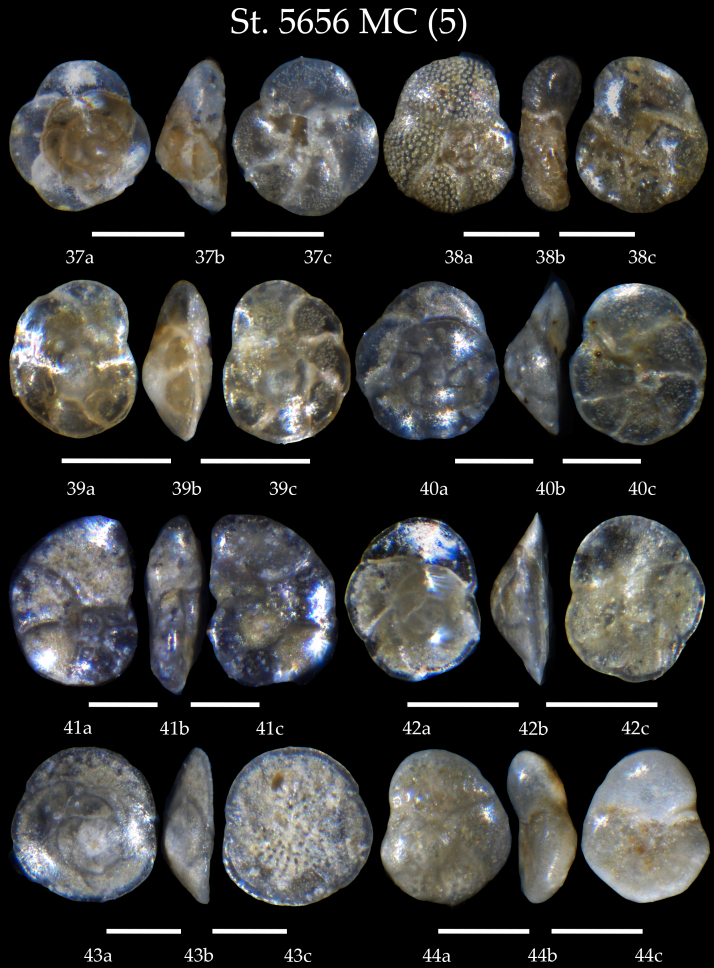
Station 5656 **MC** (continued). **37** *Rosalinavilardeboana*, **a** spiral view, **b** apertural view, **c** umbilical view; **38** *Rosalinabradyi*, **a** spiral view, **b** apertural view, **c** umbilical view; **39** *Rosalinaaraucana*, **a** spiral view, **b** apertural view, **c** umbilical view; **40** *Rosalinaauberii*, **a** spiral view, **b** apertural view, **c** umbilical view; **41** *Lamarckinahaliotidea*, **a** spiral view, **b** apertural view, **c** umbilical view; **42** *Rosalinabertheloti*, **a** spiral view, **b** apertural view, **c** umbilical view; **43** *Rosalinaopercularis*, **a** spiral view, **b** apertural view, **c** umbilical view; **44** *Rosalinaglobularis*, **a** spiral view, **b** apertural view, **c** umbilical view. Scale 100 µm.

**Figure 7.**
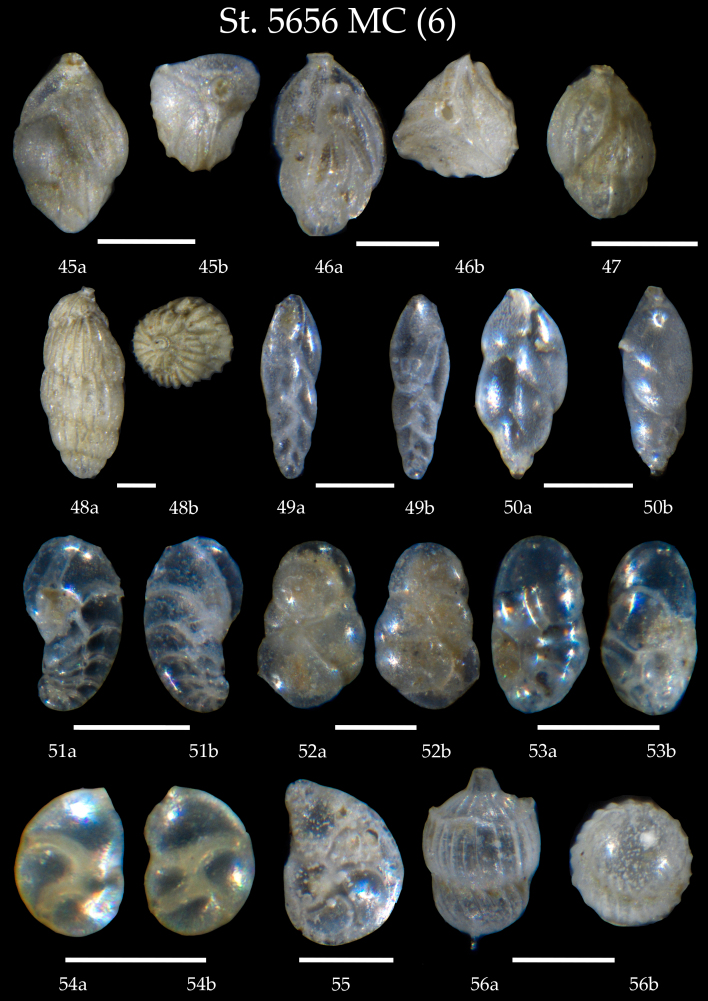
Station 5656 **MC** (continued). **45** *Trifarinaangulosa*, **a** side view, **b** apertural view; **46** *Trifarinabradyi*, **a** side view, **b** apertural view; **47** Uvigerinamediterranea; **48** *Uvigerinaperegrina*, **a** side view, **b** apertural view; **49** *Fursenkoinafusiformis*, **a** apertural view, **b** side view; **50** *Fursenkoinacomplanata*, **a** apertural view, **b** side view; **51** *Geminospirabradyi*, **a** apertural view, **b** side view; **52** *Robertinoides* sp., **a** side view, **b** lateral view; **53** *Robertinoidesbradyi*, **a** apertural view, **b** side view; **54** *Neolenticulinavariabilis*, **a** side view, **b** lateral view; **55** *Lenticulinagibba*; **56** *Amphicorynascalaris*, **a** side view, **b** apertural view. Scale 100 µm.

**Figure 8.**
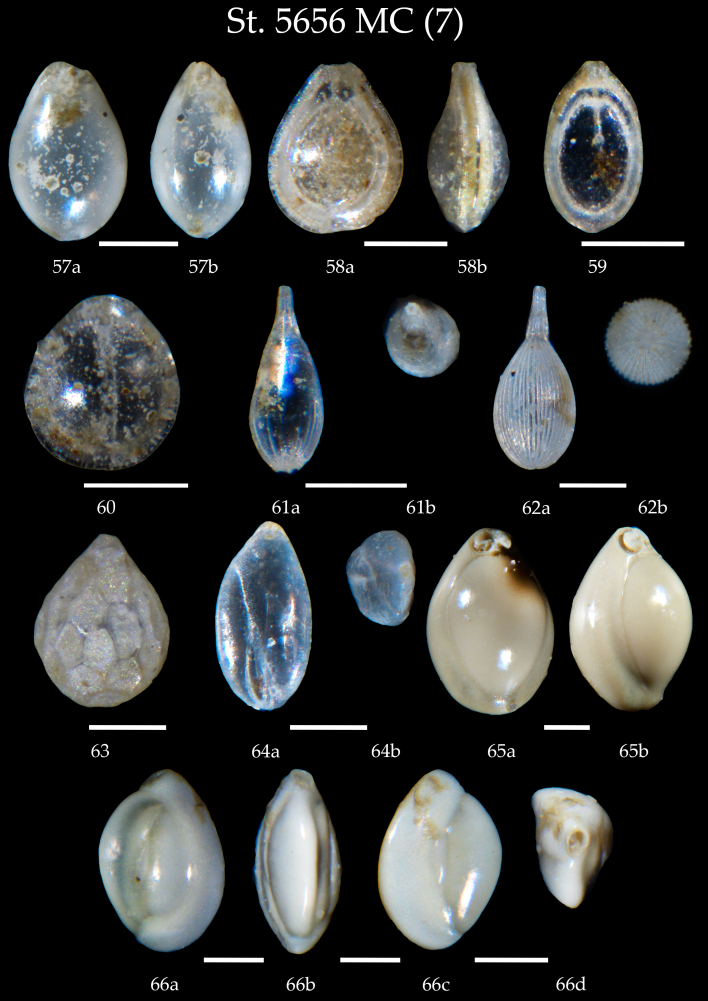
Station 5656 **MC** (continued). **57 – 60** *Fissurina* sp.; **61,62** *Lagena* sp., **a** side view, **b** apertural view; **63** *Oolina* sp.; **64** *Globobuliminapacifica*, **a** side view, **b** apertural view; **65** *Pyrgowilliamsoni*, **a** apertural view, **b** side view; **66** *Quinqueloculinaseminulum*, **a-c** side view, **d** apertural view. Scale 100 µm.

**Figure 9.**
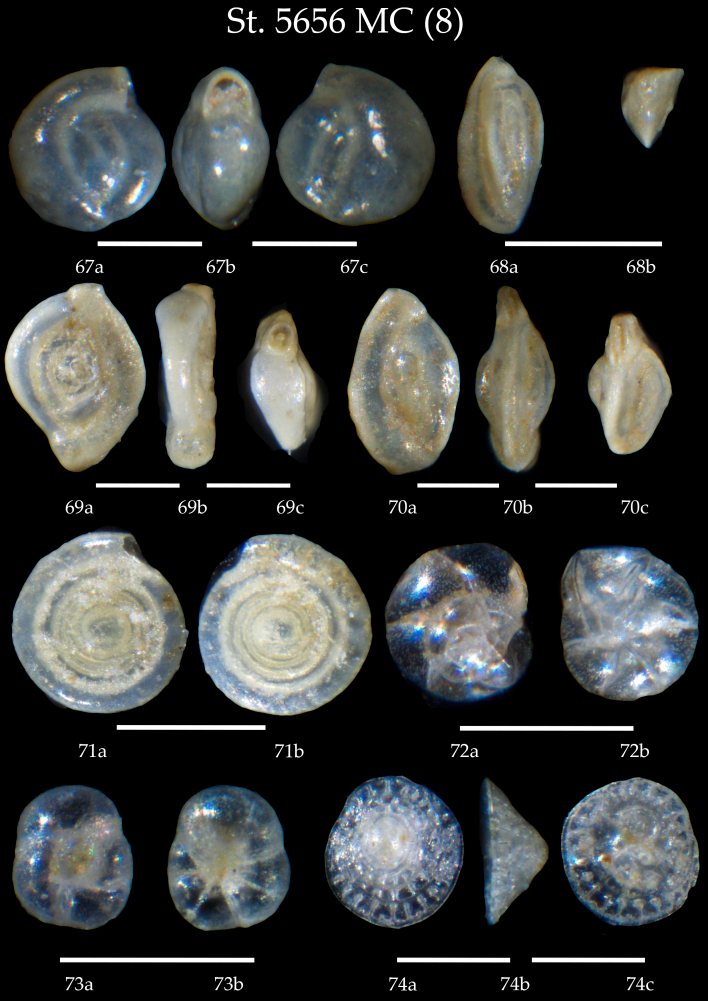
Station 5656 **MC** (continued). **67** *Miliolinellasubrotunda*, **a** side view, **b** apertural view, **c** lateral view; **68** *Triloculinaelongata*, **a** side view, **b** apertural view; **69** *Spiroloculinaexcavata*, **a, b** side view, **c** apertural view; ***70*** *Massilinasecans*, **a, b** side view, **c** apertural view; **71** *Cornuspirainvolvens*, **a** side view, **b** lateral view; **72** *Epistominellaexigua*, **a** spiral view, **b** umbilical view; **73** *Glabratellaaltispira*, **a** spiral view, **b** umbilical view; **74** *Patellinacorrugate*, **a** spiral view, **b** apertural view, **c** umbilical view. Scale 100 µm.

**Figure 10.**
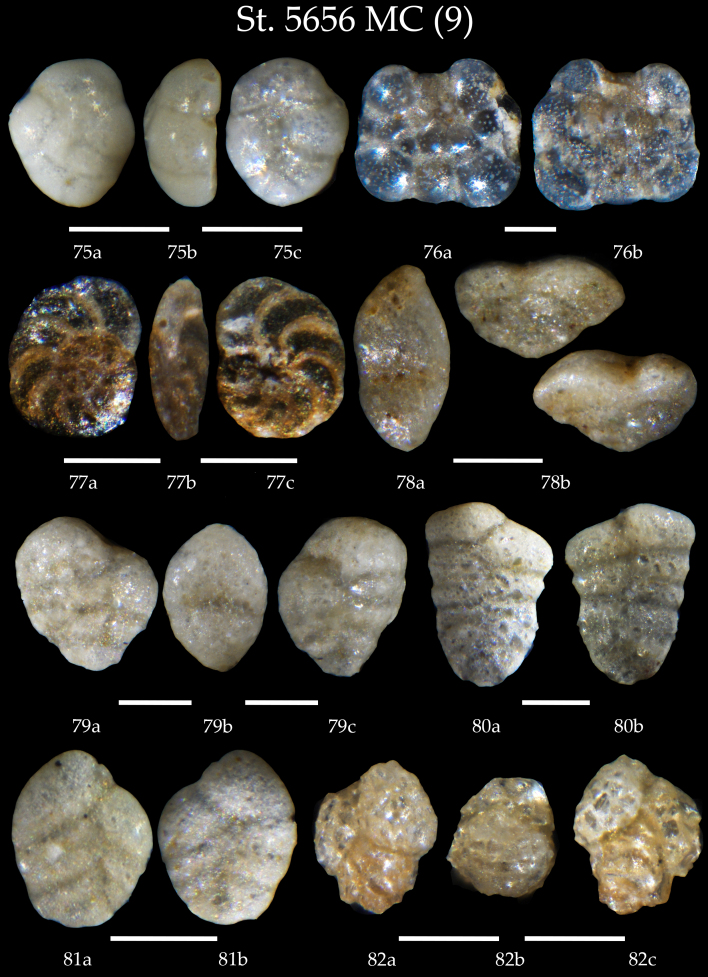
Station 5656 **MC** (continued). **75** Sp. cf. *C.lobatulus* juvenile test, **a** spiral view, **b** apertural view, **c** umbilical view; **76** *Planorbulinamediterranensis*, **a** spiral view, **b** umbilical view; **77** *Discorbis* sp., **a** spiral view, **b** apertural view, **c** umbilical view; **78** *Sahuliaconica*, **a** apertural view, **b** side view; **79** *Sahuliaconica*, **a, c** side view, **b** apertural view; **80** *Textulariasagittula*, **a** side view, **b** lateral view; **81** *Siphotextulariaconcava*, **a** side view, **b** lateral view; **82** *Eggerelloidesscaber*, **a, c** side view, **b** apertural view. Scale 100 µm.

**Figure 11.**
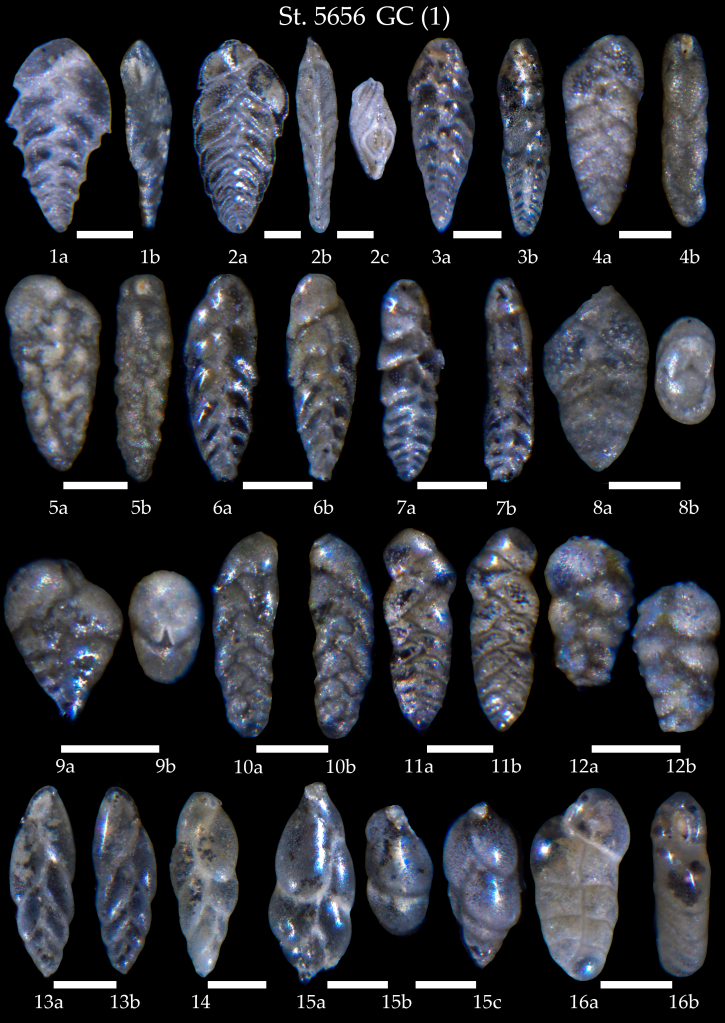
Station 5656 **GC**. **1** *Brizalinapygmaea*, **a** side view, **b** apertural view; **2** *Brizalinaalata*, **a, b** side view, **c** apertural view; **3** *Bolivinaspathulata*, **a** side view, **b** apertural view; **4** *Bolivinastriatula*, **a** side view, **b** apertural view; **5** *Bolivinapseudoplicata*, **a** side view, **b** apertural view; **6** *Bolivinaearlandi*, **a** side view, **b** lateral view; **7** *Bolivinellinapseudopunctata*, **a** side view, **b** apertural view; **8** *Bolivinaalbatrossi*, **a** side view, **b** apertural view; **9** *Bolivinainflata*, **a** side view, **b** apertural view; **10** *Bolivinalimbata*, **a** side view, **b** lateral view; **11** *Bolivina* sp., **a** side view, **b** lateral view; **12** *Bolivinasubspinescens*, **a** side view, **b** lateral view; **13,14** *Fursenkoinacomplanata*, **13a** apertural view, **13b** side view; **15** *Fursenkoinafusiformis*, **a** side view, **b, c** apertural view; **16** *Fursenkoinatexturata*, **a** side view, **b** apertural view. Scale 100 µm.

**Figure 12.**
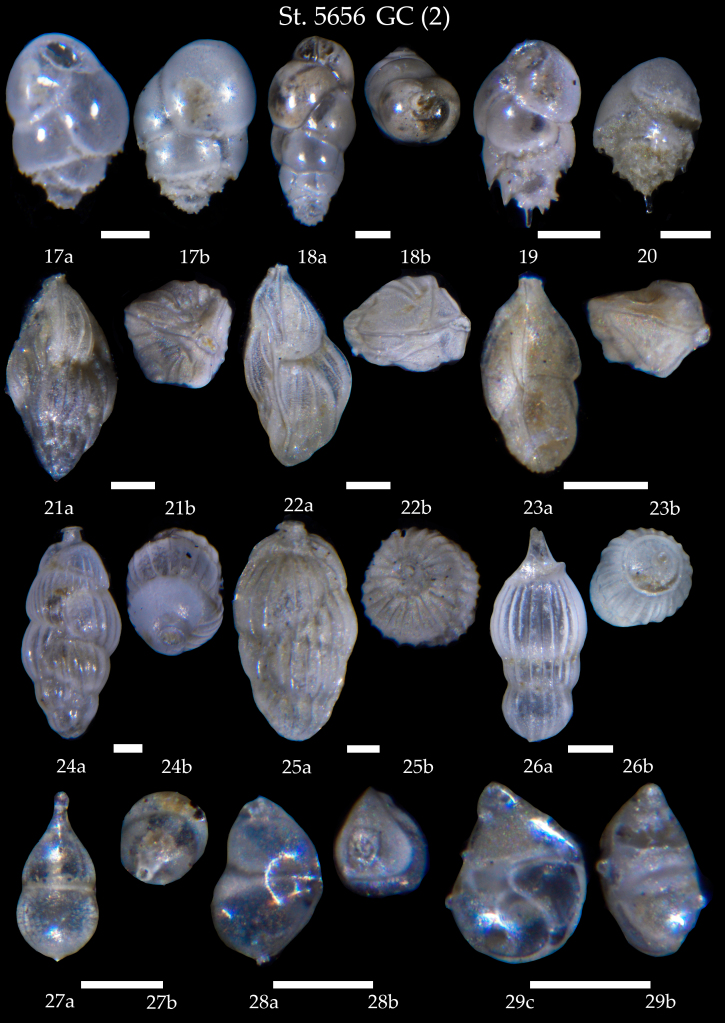
Station 5656 **GC** (continued). **17** *Buliminamarginata*, **a** side view, **b** apertural view; **18** *Buliminaelongata*, **a** side view, **b** apertural view; **19** *Buliminaaculeata*, **a** side view, **b** apertural view; **20** *Buliminaaculeata*, **a** side view, **b** apertural view; **21** *Trifarinaangulosa*, **a** side view, **b** apertural view; **22** *Trifarinafluens*, **a** side view, **b** apertural view; **23** *Trifarinabradyi*, **a** side view, **b** apertural view; **24** *Uvigerinaperegrina*, **a** side view, **b** apertural view; **25** *Uvigerinamediterranea*, **a** side view, **b** apertural view; **26** *Amphicorynascalaris*, **a** side view, **b** apertural view; **27** *Amphicorynaseparans*, **a** side view, **b** apertural view; **28** *Neolenticulinavariabilis*, **a** side view, **b** apertural view; **29** *Lenticulinagibba*, **a** side view, **b** apertural view. Scale 100 µm.

**Figure 13.**
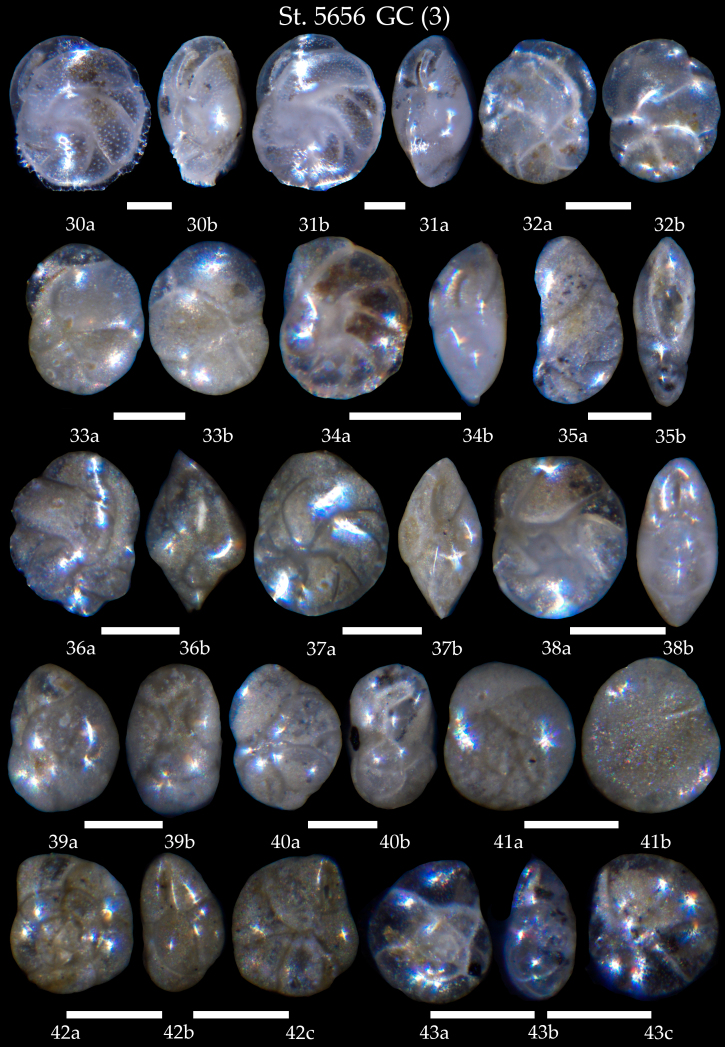
Station 5656 **GC** (continued). **30** *Cassidulinacarinata*, **a** side view, **b** apertural view; **31** *Cassidulinalaevigata*, **a** side view, **b** apertural view; **32** *Cassidulinaobtusa*, **a** apertural view, **b** lateral view; **33** *Cassidulinareniforme*, **a** apertural view, **b** lateral view; **34** *Cassidulinateretis*, **a** side view, **b** apertural view; **35** *Cassidulinoidesbradyi*, **a** side view, **b** apertural view; **36,37** *Cassidulina* sp. cf *C.laevigata* **a** side view, **b** apertural view; **38** *Islandiellanorcrossi*, **a** side view, **b** apertural view; **39,40** *Globocassidulinasubglobosa*, **a** side view, **b** apertural view; **41** *Buccellafrigida*, **a** spiral view, **b** umbilical view; **42** *Epistominellavitrea*, **a** spiral view, **b** apertural view, **c** umbilical view; **43** *Epistominellaexigua*, **a** spiral view, **b** apertural view, **c** umbilical view. Scale 100 µm.

**Figure 14.**
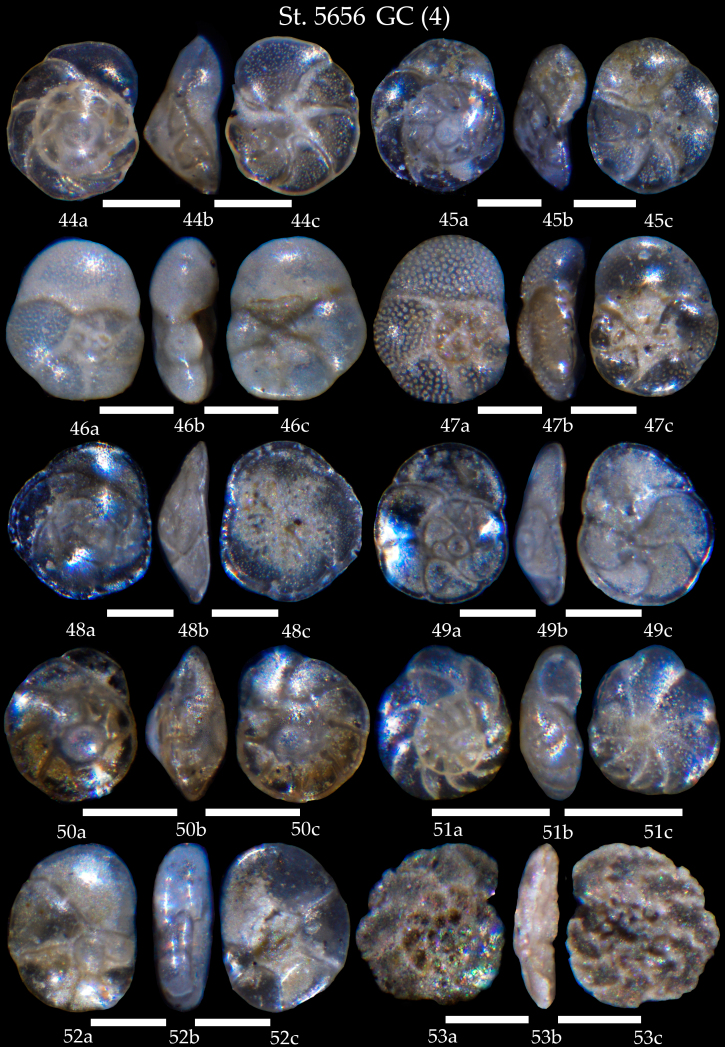
Station 5656 **GC** (continued). **44,45** *Rosalinavilardeboana*, **a** spiral view, **b** apertural view, **c** umbilical view; **46** *Rosalinaglobularis*, **a** spiral view, **b** apertural view, **c** umbilical view; **47** *Rosalinabradyi*, **a** spiral view, **b** apertural view, **c** umbilical view; **48** *Rosalinaopercularis*, **a** spiral view, **b** apertural view, **c** umbilical view; **49** *Rosalinabertheloti*, **a** spiral view, **b** apertural view, **c** umbilical view; **50** *Rosalinaaraucana*, **a** spiral view, **b** apertural view, **c** umbilical view; **51** *Rosalina* sp., **a** spiral view, **b** apertural view, **c** umbilical view; **52** *Lamarckinahaliotidea*, **a** spiral view, **b** apertural view, **c** umbilical view; **53** *Discorbis* sp., **a** spiral view, **b** apertural view, **c** umbilical view. Scale 100 µm.

**Figure 15.**
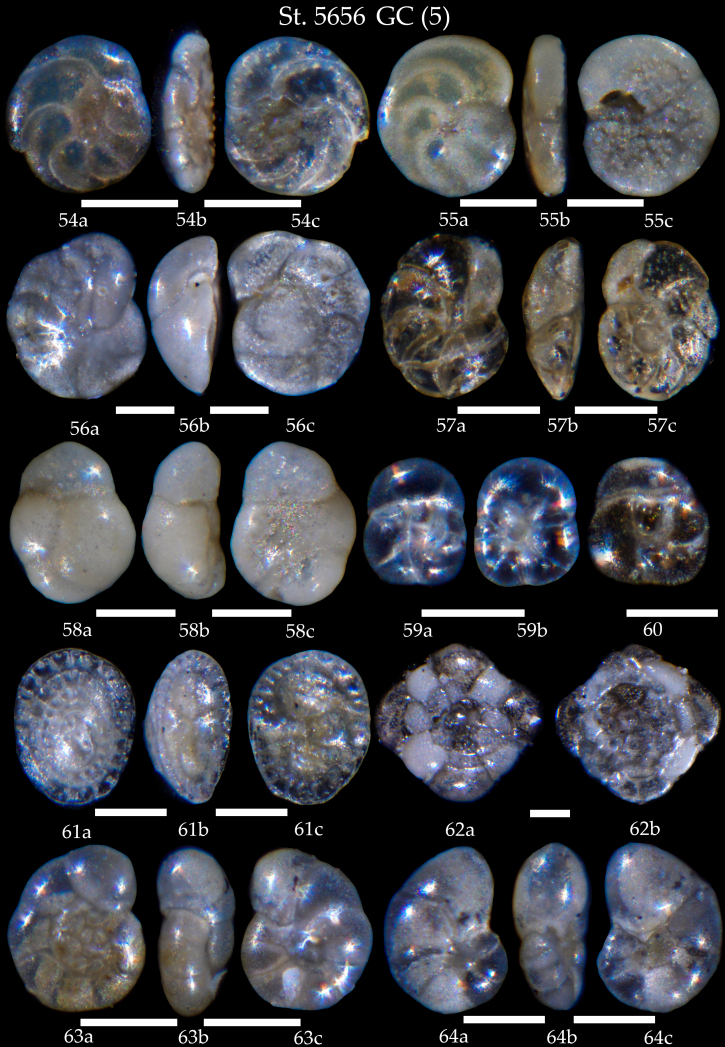
Station 5656 **GC** (continued). **54** *Discorbis* sp., **a** spiral view, **b** apertural view, **c** umbilical view; **55** *Neoglabratellawiesneri*, **a** spiral view, **b** apertural view, **c** umbilical view; **56** *Cibicideslobatulus*, **a** spiral view, **b** apertural view, **c** umbilical view; **57** *Cibicidoideswuellerstorfi*, **a** spiral view, **b** apertural view, **c** umbilical view; **58** Sp. cf. *C.lobatulus* juvenile test, **a** spiral view, **b** apertural view, **c** umbilical view; **59,60** *Glabratellaaltispira*, **59a** spiral view, **59b** umbilical view; **61** *Patellinacorrugata*, **a** spiral view, **b** apertural view, **c** umbilical view; **62** *Planorbulinamediterranensis*, **a** spiral view, **b** umbilical view; **63** *Valvulineriarugosa*, **a** spiral view, **b** apertural view, **c** umbilical view; **64** *Valvulineriaminuta*, **a** spiral view, **b** apertural view, **c** umbilical view. Scale 100 µm

**Figure 16.**
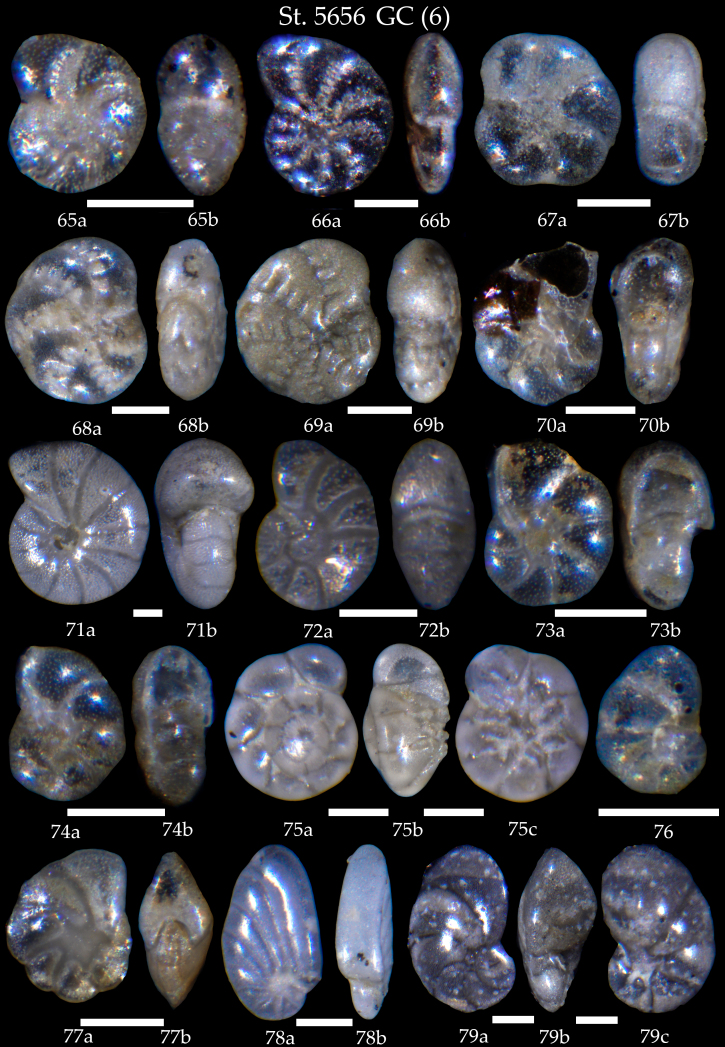
Station 5656 **GC** (continued). **65** *Elphidiumclavatum*, **a** side view, **b** apertural view; **66** *Elphidiumgerthi*, **a** side view, **b** apertural view; **67** *Elphidium* sp. cf. *E.magellanicum*, **a** side view, **b** apertural view; **68** *Elphidiumearlandi*, **a** side view, **b** apertural view; **69** *Elphidium* sp. cf. *E.williamsoni*, **a** side view, **b** apertural view; **70** *Astrononiongallowayi*, **a** side view, **b** apertural view; **71** *Melonispompilioides*, **a** side view, **b** apertural view; **72** *Melonisbarleeanus*, **a** side view, **b** apertural view; **73,74** Sp. cf. *Haynesinadepressula*, **a** side view, **b** apertural view; **75** *Ammoniafalsobeccarii*, **a** spiral view, **b** apertural view, **c** umbilical view; **76** *Nonionellaauricula*; **77** *Nonionpauperatum*, **a** side view, **b** apertural view; **78** *Nonionoidesturgidus*; **79** *Nonionellairidea*, **a** spiral view, **b** apertural view, **c** umbilical view. Scale 100 µm.

**Figure 17.**
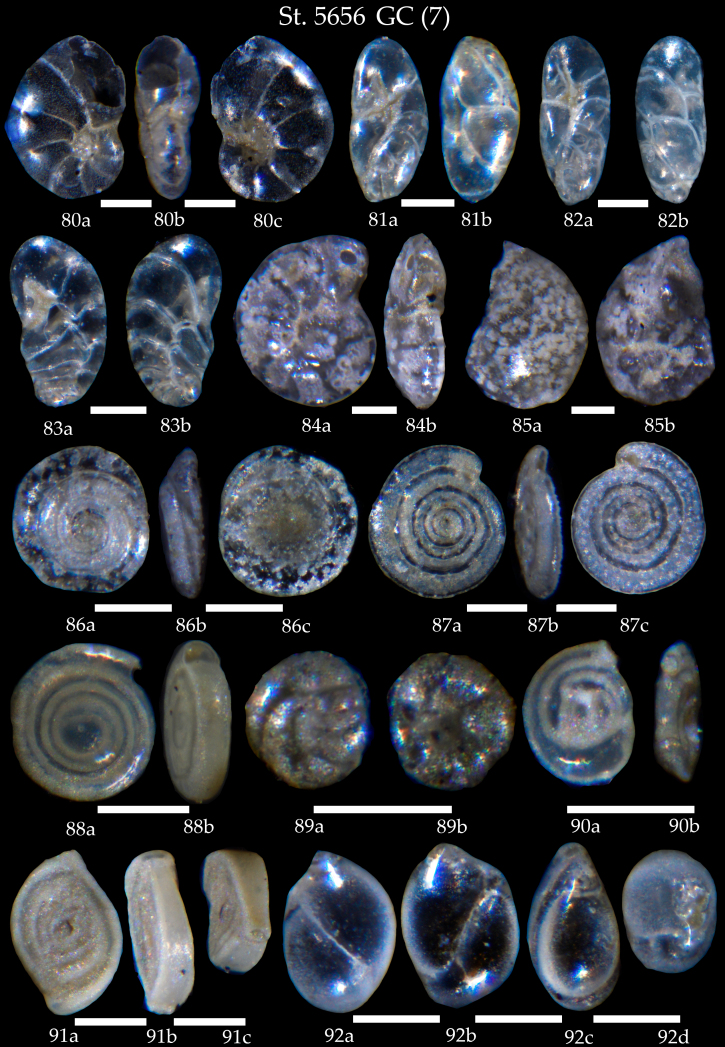
Station 5656 **GC** (continued). **80** *Nonion* sp., cf. *N.faba*, **a** spiral view, **b** apertural view, **c** umbilical view; **81,82** *Robertinoidesbradyi*, **a** apertural view, **b** lateral view; **83** *Geminospirabradyi*, **a** apertural view, **b** lateral view; **84** *Hyalineabalthica*, **a** side view, **b** apertural view; **85** Sp.; **86** *Mychostominarevertens*, **a** spiral view, **b** apertural view, **c** umbilical view; **87** *Spirillinaviviparina*, **a** spiral view, **b** apertural view, **c** umbilical view; **88** *Cornuspirainvolvens*, **a** side view, **b** apertural view; **89** *Facetocochleapulchra*, **a** spiral view, **b** umbilical view; **90** *Spiroloculina* sp., **a** side view, **b** apertural view; **91** *Spiroloculinadepressa*, **a, b** side view, **c** apertural view; **92** *Globobuliminapacifica*, **a-c** side view, **d** apertural view. Scale 100 µm.

**Figure 18.**
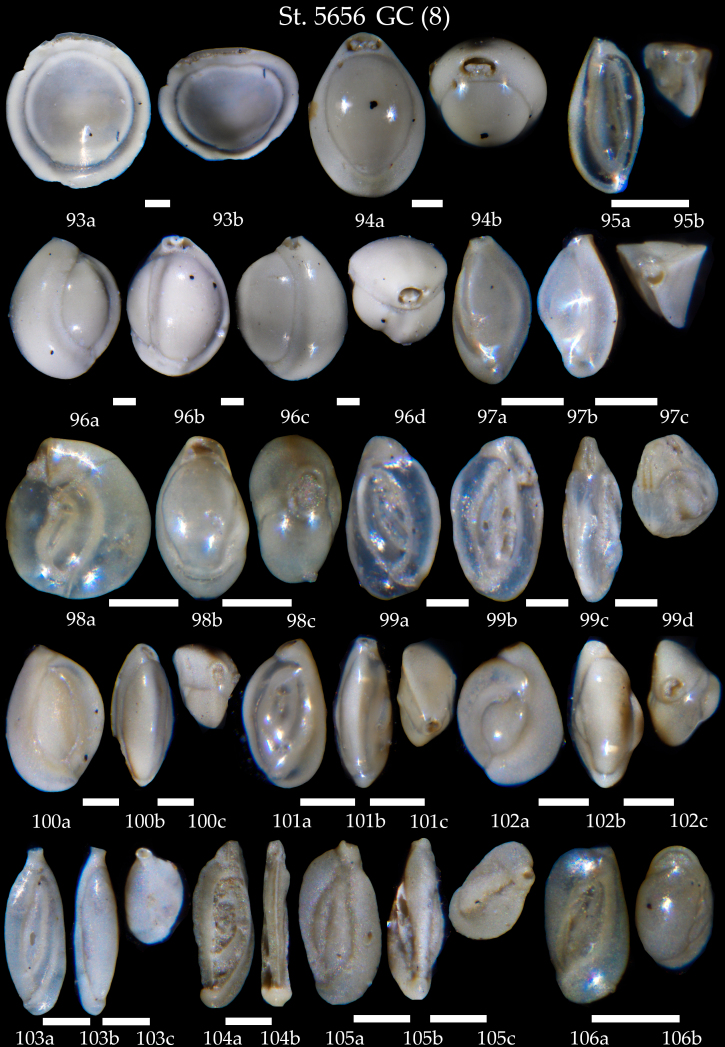
Station 5656 **GC** (continued). **93** *Pyrgomurrhina*, **a** side view, **b** apertural view; **94** *Pyrgowilliamsoni*, **a** side view, **b** apertural view; **95** *Triloculinaelongata*, **a** side view, **b** apertural view; **96** *Triloculinatrigonula*, **a-c** side view, **d** apertural view; **97** *Triloculinatrihedra*, **a, b** side view, **c** apertural view; **98** *Miliolinellasubrotunda*, **a, b** side view, **c** apertural view; **99** *Massilinasecans*, **a-c** side view, **d** apertural view; **100** *Quinqueloculinaseminulum*, **a, b** side view, **c** apertural view; **101** *Quinqueloculina* sp. cf. *Q.seminulum*; **102-106** *Quinqueloculina* sp. Scale 100 µm.

**Figure 19.**
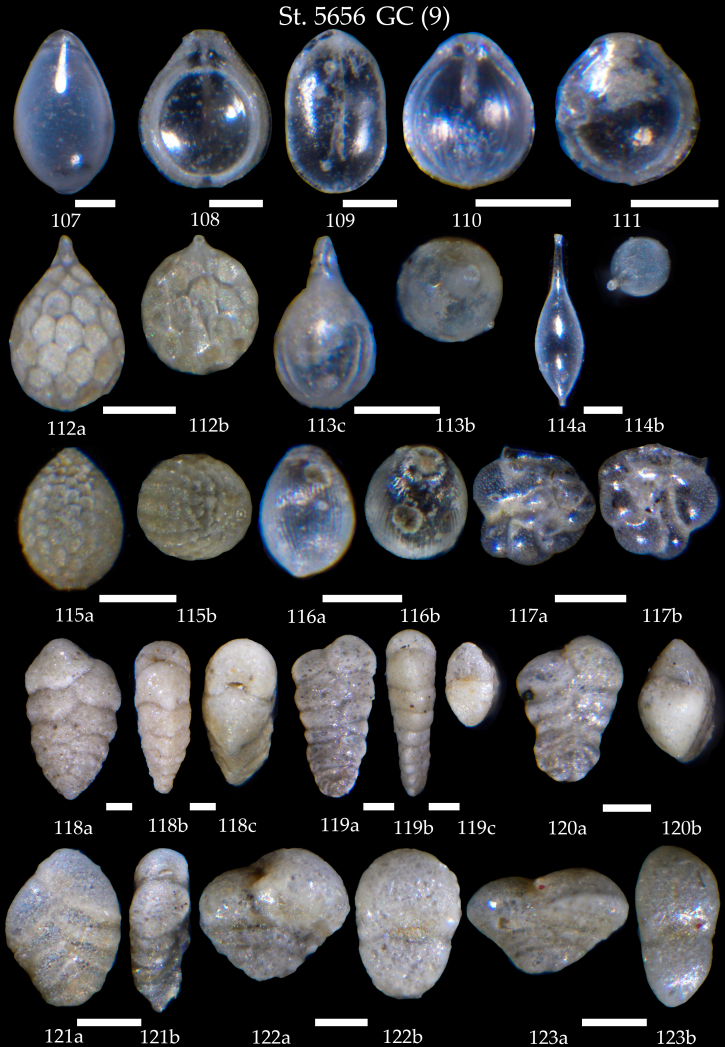
Station 5656GC (continued). **107-111** *Fissurina* sp.; **112,113** *Lagena* sp., **a** side view, **b** apertural view; **114** *Procerolagenaclavata*, **a** side view, **b** apertural view; **115,116** *Oolina* sp. **a** side view, **b** apertural view; **117** Sp. cf. *Asterorotaliapulchella*, **a, b** side view; **118** *Textulariasagittula*, **a, b** side view, **c** apertural view; **119,120** *Spiroplectinellawright*, **119a, b** side view, **119c** apertural view, **120a** side view, **120b** apertural view; **121** *Siphotextulariaconcava*, **a, b** side view, **b** apertural view; **122,123** *Sahuliaconica*, **a** side view, **b** apertural view. Scale 100 µm.

**Table 1.** List of benthic foraminiferal taxa in station AMK-5656 from north-western Scotland shelf in North Atlantic.

*Ammoniafalsobeccarii* (Rouvillois, 1974)
*Amphicorynascalaris* (Batsch, 1791)
*Amphicorynaseparans* (Brady, 1884)
*Astrononiongallowayi* Loeblich & Tappan, 1953 = *Astrononionhamadaense* Asano, 1950
*Bolivinaalbatrossi* Cushman, 1922
*Bolivinaearlandi* Parr, 1950
*Bolivinainflata* Heron-Allen & Earland, 1913
*Bolivinalimbata* (Brady, 1881) = *Loxostominalimbata* (Brady, 1881)
*Bolivinapseudoplicata* Heron-Allen & Earland, 1930
*Bolivinaspathulata* (Williamson, 1858)
*Bolivinastriatula* Cushman, 1922
*Bolivinasubspinescens* Cushman, 1922
*Bolivinatortuosa* (Brady, 1881) = *Sigmavirgulinatortuosa* (Brady, 1881)
*Bolivina* sp. d'Orbigny, 1839
*Bolivinellinapseudopunctata* (Höglund, 1947)
*Brizalinaalata* (Seguenza, 1862) = *Bolivinaalata* (Seguenza, 1862)
*Brizalinapygmaea* (Brady, 1881) = *Bolivinapygmaea* (Brady, 1881)
*Buccellafrigida* (Cushman, 1922) = *Buccellacalida* (Cushman & Cole, 1930)
*Buliminaaculeata* d'Orbigny, 1826
*Buliminaelongata* d'Orbigny, 1846
*Buliminamarginata* d'Orbigny
*Cassidulinacarinata* Silvestri, 1896
*Cassidulinalaevigata* d'Orbigny, 1826
*Cassidulinaobtusa* Williamson, 1858
*Cassidulinareniforme* Nørvang, 1945
*Cassidulinateretis* Tappan, 1951 = *Cassidulinaneoteretis* Seidenkrantz, 1995
*Cassidulina* sp. d'Orbigny, 1826
*Cassidulinoidesbradyi* (Norman, 1881)
*Cornuspirainvolvens* (Reuss, 1850)
*Cibicidesrefulgens* Montfort, 1808
*Cibicideslobatulus* (Walker & Jacob, 1798) = *Lobatulalobatula* (Walker & Jacob, 1798)
*Cibicidoideswuellerstorfi* (Schwager, 1866)
*Cibicides* sp. Montfort, 1808
*Discorbis* sp. Lamarck, 1804
*Eggerelloidesscaber* (Williamson, 1858)
*Elphidiumclavatum* Cushman, 1930 = Elphidiumexcavatumsubsp.clavatum Cushman, 1930
*Elphidiumearlandi* Cushman, 1936
*Elphidiumgerthi* van Voorthuysen, 1957 = *Cribroelphidiumgerthi* (van Voorthuysen, 1957)
*Elphidium* sp. Montfort, 1808
*Epistomaroidespolystomelloides* (Parker & Jones, 1865)
*Epistominellaexigua* (Brady, 1884)
*Epistominellavitrea* Parker, 1953 = *Eilohedravitrea* (Parker, 1953)
*Facetocochleapulchra* (Cushman, 1933)
*Fissurina* sp. Reuss, 1850
*Fursenkoinafusiformis* (Williamson, 1858) = *Stainforthiafusiformis* (Williamson, 1858)
*Fursenkoinacomplanata* (Egger, 1893) = *Stainforthialoeblichi* (Feyling-Hanssen, 1954)
*Fursenkoinatexturata* (Brady, 1884)
*Geminospirabradyi* Bermúdez, 1952
*Glabratellaaltispira* Buzas, Smith & Beem, 1977
*Globobuliminapacifica* Cushman, 1927 = *Laryngosigmalactea* (Walker & Jacob, 1798)
*Globocassidulinasubglobosa* (Brady, 1881)
*Hyalineabalthica* (Schröter, 1783)
*Islandiellanorcrossi* (Cushman, 1933)
*Lagena* sp. Walker & Jacob, 1798
*Lamarckinahaliotidea* (Heron-Allen & Earland, 1911)
*Lenticulinagibba* (d'Orbigny, 1839)
*Massilinasecans* (d'Orbigny, 1826) = *Quinqueloculinasecans* d'Orbigny, 1826
*Melonisbarleeanus* (Williamson, 1858) = *Melonisaffinis* (Reuss, 1851)
*Melonispompilioides* (Fichtel & Moll, 1798)
*Melonis* sp. Montfort, 1808
*Miliolinellasubrotunda* (Montagu, 1803)
*Mychostominarevertens* (Rhumbler, 1906)
*Neoglabratellawiesneri* (Parr, 1950)
*Neolenticulinavariabilis* (Reuss, 1850)
*Nonionpauperatum* (Balkwill & Wright, 1885) = *Subanomalinapauperata* (Balkwill & Wright, 1885)
*Nonion* sp. Montfort, 1808
*Nonionellaauricula* Heron-Allen & Earland, 1930
*Nonionellairidea* Heron-Allen & Earland, 1932
*Nonionoidesturgidus* (Williamson, 1858)
*Oolina* sp. d'Orbigny, 1839
*Patellinacorrugata* Williamson, 1858
*Planorbulinamediterranensis* d'Orbigny, 1826
*Planulina* sp. d'Orbigny, 1826
*Procerolagenaclavata* (d'Orbigny, 1846)
*Pyrgomurrhina* (Schwager, 1866)
*Pyrgowilliamsoni* (Silvestri, 1923)
*Quinqueloculinaseminulum* (Linnaeus, 1758)
*Quinqueloculina* sp. d'Orbigny, 1826
*Robertinoidesbradyi* (Cushman & Parker, 1936)
*Robertinoides* sp. Höglund, 1947
*Rosalinaaraucana* d'Orbigny, 1839 = *Valvulineriaaraucana* (d'Orbigny, 1839)
*Rosalinaauberii* d'Orbigny, 1839 = *Discorbisauberii* (d'Orbigny, 1839) = *Rotorbisauberii* (d'Orbigny, 1839)
*Rosalinabertheloti* d'Orbigny, 1839 = *Discorbinellabertheloti* (d'Orbigny, 1839)
*Rosalinabradyi* (Cushman, 1915) = *Rosalinaanomala* Terquem, 1875
*Rosalinaglobularis* d'Orbigny, 1826
*Rosalinaopercularis* d'Orbigny, 1826 = *Rosalinaopercularis* d'Orbigny, 1839 = *Discorbinaopercularis* (d'Orbigny, 1839)
*Rosalinavilardeboana* d'Orbigny, 1839 = *Discorbisvilardeboanus* (d'Orbigny, 1839)
*Rosalina* sp. d'Orbigny, 1826
*Sahuliaconica* (d'Orbigny, 1839) = *Textulariaconica* d'Orbigny, 1839
*Siphotextulariaconcava* (Karrer, 1868)
*Spiroloculinadepressa* d'Orbigny, 1826
*Spiroloculinaexcavata* d'Orbigny, 1846
*Spiroloculina* sp. d'Orbigny, 1826
*Spirillinaviviparina* Saidova, 1975
*Spiroplectinellawrighti* (Silvestri, 1903)
*Textulariasagittula* Defrance, 1824
*Triloculinaelongata* d'Orbigny in Fornasini, 1905
*Triloculinatrigonula* (Lamarck, 1804)
*Triloculinatrihedra* Loeblich & Tappan, 1953
*Trifarinaangulosa* (Williamson, 1858)
*Trifarinabradyi* Cushman, 1923
*Trifarinafluens* (Todd in Cushman & McCulloch, 1948)
*Uvigerinaperegrina* Cushman, 1923
*Uvigerinamediterranea* Hofker, 1932
*Valvulineriaminuta* (Schubert, 1904) = *Discorbisminuta* (Schubert, 1904)
*Valvulineriarugosa* (d'Orbigny, 1839) = *Discorbisrugosa* (d'Orbigny, 1839) = *Rosalinarugosa* d'Orbigny, 1839
